# Effect size of rituximab on pulmonary function in the treatment of connective-tissue disease-related interstitial lung disease: a systematic review and meta-analysis

**DOI:** 10.1186/s12931-022-02082-x

**Published:** 2022-06-21

**Authors:** Yuanchen Zhao, Yang Gao, Tananchai Petnak, Wisit Cheungpasitporn, Charat Thongprayoon, Xing Zhang, Teng Moua

**Affiliations:** 1 Division of Pulmonary Guang’anmen Hospital, China Academy of Chinese Medical Sciences, No. 5, Beixiange, Xicheng, Beijing, China; 2 Division of Pulmonary and Pulmonary Critical Care Medicine, Faculty of Medicine Ramathibodi Hospital, Mahidol University, Salaya, Thailand; 3 Division of Nephrology and Hypertension, Mayo Clinic, Rochester, MN USA; 4 Division of Pulmonary and Critical Care Medicine, Mayo Clinic, 200 First St SW, Rochester, MN 55905 USA

**Keywords:** Rituximab, Connective-tissue disease related interstitial lung disease, Meta-analysis, Systematic review

## Abstract

**Background:**

Rituximab (RTX) has been previously reported as directed treatment in patients with connective-tissue disease-related interstitial lung diseases (CTD-ILD). A systematic assessment of treatment effect size on pulmonary function outcomes and related adverse effects in patients with CTD-ILD has not been previously reported.

**Methods:**

We performed a systematic review and meta-analysis of published reports from PubMed, Embase, and Cochrane Libraries. Randomized and non-randomized controlled trials, case–control, cohort, and case series (with five or more cases) containing individual pulmonary function data and adverse effects were included. Study endpoints were pre- and post-treatment change in percent predicted forced vital capacity (FVC %) and diffusion capacity for carbon monoxide (DLCO%), along with reported drug-related adverse events.

**Results:**

Twenty studies totaling 411 patients were identified with 14 included in the meta-analysis of pulmonary function and six in the descriptive review. Random effects meta-analysis of pre- and post-treatment pulmonary function findings demonstrated increases in FVC% (n = 296) (mean difference (MD) 4.57%, [95% CI 2.63–6.51]) and DLCO% (n = 246) (MD 5.0% [95% CI 2.71–7.29]) after RTX treatment. RTX treatment-related adverse effects were reported in 13.6% of the pooled cohort.

**Conclusions:**

A systematic assessment of post-treatment effect size suggests a potential role for RTX in stabilizing or improving lung function in patients with CTD-ILD, with a modest but not insignificant adverse effect profile.

**Supplementary Information:**

The online version contains supplementary material available at 10.1186/s12931-022-02082-x.

## Introduction

The connective-tissue diseases (CTD) are commonly associated with initial or subsequent interstitial lung disease, frequently portending greater morbidity than CTD without lung involvement [1]. While nearly all CTD may be associated with ILD, systemic sclerosis (SSc), the idiopathic inflammatory myopathies (IIM), and rheumatoid arthritis (RA) report the highest prevalences [2–4]. Radiologic patterns and clinical manifestations vary according to subtype while pulmonary function is frequently characterized by restrictive ventilatory defect and reduced diffusing capacity[5]. Therapy for the majority of CTD-ILD often involves an extension of medications already aimed at the underlying CTD, typically consisting of corticosteroids and steroid-sparing agents like cyclophosphamide (CYC), azathioprine (AZA), and mycophenolate mofetil (MMF) [3]. Except for scleroderma-ILD, management of CTD-ILD remains experiential or empiric due to lack of robust randomized controlled trials (RCT). Heterogeneity of disease subtypes, unclear and variable outcome measures, and relatively better survival compared to other progressive ILD like idiopathic pulmonary fibrosis (IPF) make large controlled studies difficult, requiring expansive RCTs with longer durations to differentiate functional or survival outcomes. Pulmonary function endpoints are often followed as reasonable markers of treatment effect.

Rituximab (RTX), a B-cell depleting chimeric monoclonal antibody against human CD20, is currently approved for the treatment of lymphoma and RA. With prior evidence suggesting aberrations in lymphocyte function may be involved in the development and evolution of CTD [6], its use for the treatment of other CTD subtypes has gained recent interest. An initial report in 2008 involving patients with SSc-ILD [7] supported its particular role in CTD with associated ILD, particularly those with clinically severe or progressive lung disease unresponsive to conventional immunosuppression [8]. Multiple case reports, case series, and one clinical trial have reported on the positive effects of rituximab in CTD-ILD though the extent of its effect on measured pulmonary function has not been summatively reported. This systematic review and meta-analysis summarizes the pooled effect size of RTX on lung function (percent predicted forced vital capacity (FVC%) and diffusion capacity for carbon monoxide (DLCO%)) and describes reported safety outcomes in the treatment of CTD-ILD.

## Materials and methods

The current systematic review and meta-analysis was conducted and reported according to the Preferred Reporting Items for Systematic reviews and Meta-Analyses (PRISMA) [9] guideline and statement. The Cochrane Handbook for Systematic Reviews of Interventions [10] provided the methodology for meta-analysis, and specific scoring of the included citations. The protocol was registered with ResearchRegistry (www.researchregistry.com; identifier reviewregistry1182).

### Literature search and study selection

PubMed, Cochrane Library, and Embase databases were searched for original full articles published in English from their inception to March 20, 2021. Search terms, combinations, and results are presented in Additional file 1: Table S1. Studies were selected from case series reporting five or more cases, case–control, retrospective or prospective cohorts, and randomized or non-randomized controlled trials. Only adults (18 to 80 years of age) with a diagnosis of CTD-ILD were included. In terms of intervention, RTX was used individually or in combination with other immunosuppressive agents for at least six months or more (one cycle). Two reviewers (YZ and YG) assessed the titles and abstracts of all search results with pre-specified inclusion and exclusion criteria. If the abstract of an article suggested relevance, the article was retrieved and independently assessed by the same reviewers for inclusion in either the descriptive review or meta-analysis of pulmonary function outcomes.

### Risk of bias assessment and overall quality of evidence

Risk of bias was assessed according to study type by the following: Cochrane collaboration tool for bias assessment in randomized controlled trials; Joanna Briggs Institute (JBI) critical appraisal checklist for quasi-experimental studies for nonrandomized observational studies; JBI critical appraisal checklist for cohort studies; and the JBI critical appraisal checklist for case series [10, 11]. Publication bias was assessed by funnel plot and Egger test.

### Data extraction

Pre-specified study data included design, duration of follow-up, setting, and performance dates. Participant data included number, mean age and age range, sex, and CTD-ILD subtype. Intervention data included RTX dose and number and concomitant immunosuppression. Adverse events or complications attributed to RTX were also collated and categorized. Disagreements regarding study inclusion were resolved by consensus between study reviewers (YZ and YG).

### Endpoints

Primary meta-analysis outcomes were mean differences (MD) in percent predicted pre- and post-treatment reported forced vital capacity (FVC%) and diffusion capacity for carbon monoxide (DLCO%). Included articles for meta-analysis required reporting of specific pre- and post-treatment lung function findings or the mean changes in FVC% or DLCO% from baseline and their calculated standard deviations. Post-treatment FVC% and DLCO% were defined as obtained six months or greater from the first dose of RTX. All RTX dosages and infusion regimens were included, with all patients treated for at least one course of treatment (6 months). Drug-related adverse events from all studies were reviewed.

### Data analysis

Meta-analysis was performed using Comprehensive Meta-Analysis software version 3.3.070 (Biostat Inc, Englewood, NJ, USA). Descriptive analysis was conducted separately for non-combined studies (n = 6). Mean and standard deviation (SD) for changes in FVC% and DLCO% were calculated using the following equations from the Cochrane Handbook for Systematic Reviews of Interventions [10]:

$${\text{Mean}}\;{\mkern 1mu} {\text{change}}\;{\mkern 1mu} {\text{from}}\;{\mkern 1mu} {\text{baseline}} = {\text{Mean}}_{{{\text{post-treatment}}}} - {\text{Mean}}_{{{\text{pre-treatment}}}}$$

$${\mathrm{SD}}_{\mathrm{change}}= \sqrt{{{\mathrm{SD}}_{\mathrm{baseline}}}^{2}+ {{\mathrm{SD}}_{\mathrm{final}}}^{2}-(2 \times \mathrm{r }\times {\mathrm{SD}}_{\mathrm{baseline}} \times {\mathrm{SD}}_{\mathrm{final}})}$$

R in the equation above represents a correlation coefficient for which we imputed values of 0.4, 0.6, and 0.8 with sensitivity analysis for each. As the pooled MD results were similar for each value, we used a coefficient of 0.4 for r in the meta-analysis. MD for FVC% and DLCO% were reported as mean changes from baseline with 95% CI. A random-effects model with DerSimonian-Laird estimator of between-study variance was used to estimate final MD for each endpoint given baseline differences in study type and patient characteristics [12]. Magnitude of treatment effect may also vary according to sample size, disease subtype, concomitant treatment, or other unaccounted covariables, with a random effects model assuming effect size may be similar but not identical across all included studies with the intent of reporting the pooled true effects. The *I*^*2*^ statistic was used to describe heterogeneity among the studies (*I*^*2*^ > 50% or *P* value < 0.10 for high heterogeneity). Meta-analysis data are presented as summary Forest plots for each PFT endpoint.

## Results

### Study selection and patient characteristics

**Fig. 1:**
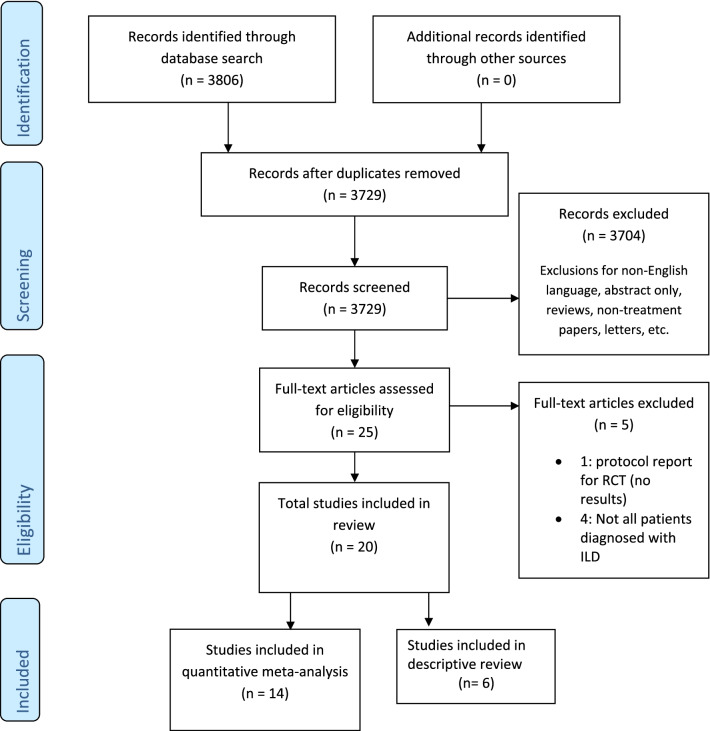
PRISMA study selection

A total of 3806 individual citations were found and screened, resulting in the inclusion of 20 studies for systematic review, 14 of which were included specifically in the quantitative meta-analysis of treatment effect size on lung function [13–32]. Study selection is presented in Fig. 1. Study exclusion on initial screening included non-English language, reporting of non-functional outcomes, reviews, editorials or commentary, incomplete manuscripts, lay press, and case reports with less than five cases. Tables 1 and 2 summarize study characteristics included in the meta-analysis (n = 14) and descriptive review (n = 6). Sample sizes ranged from seven to 56, totaling 411 patients. Study subtypes included case series (n = 13), retrospective cohorts (n = 5), one non-randomized trial, and one RCT. Six studies reported the combined outcomes of several CTD-ILD; five for SSc-ILD and antisynthetase syndrome-ILD (ASS-ILD); three for RA-ILD, and one for primary Sjögren’s-related-ILD (pSS). Summarized patient characteristics for the meta-analysis and descriptive reviews are presented in Table 3). There was female predominance for both analyses with a majority being treated for progressive ILD dominated by fibrosis unresponsive to initial immunosuppression. Thirty-three patients in one intervention study were randomized to RTX [21] as rationale for initiation with only ten patients from both analyses being provided RTX as first-line therapy [20, 33]. In most studies, RTX was administered as two 1000 mg infusions two weeks apart, or 375 mg/m^2^ weekly for 4 weeks. Each cycle could be repeated at six-month intervals with all included studies reporting on the outcomes of at least one treatment cycle. Steroid-sparing immunosuppressant and other therapies included AZA, MMF, CYC, methotrexate (MTX) and intravenous immunoglobulin (IVIG). Pulmonary function outcomes were measured at baseline and after at least a first RTX treatment period (six months) up to one year.

**Table 1:** Characteristics of studies included in the meta-analysis (n = 14 studies)

Author and year	CTD-ILD subtype	Study design	N	RTX Dose	Duration	Concurrent treatment	Outcomes	Duration of follow-up	Adverse events with RTX
Daoussis et al. 2012	SSc-ILD	Case series	8	One cycle: IV, 375 mg/ m^2^, once weekly*4 weeks, every 6 months	4 cycles	None	FVC; DLCO	2 years	2 Respiratory infections requiring hospitalization, 1 with associated leukopenia, 1 infusion reaction
Keir et al. 2012	CTD-ILD	Case series	6	One cycle: 5-IV, 1000 mg administered on day 0 and day 14 1 patient: 375 mg/ m^2^, once weekly*4 weeks	1 cycle	None	FVC; DLCO	9–12 months	None
Fitzgerald et al. 2015	CTD-ILD	Case series	10	a. 1–1000 mg monthly b. 2-IV, 375 mg/ m^2^, monthly c. 7-IV, 1000 mg administered on day 0 and day 14	a, b*4 months; c*6 months	CYC*3	FVC; DLCO	3–27 months	None
Chen et al. 2016	Sjögren’s syndrome-ILD	Case series	10	One cycle: IV, 1000 mg administered on day 0 and day 14	Repeated the same protocol every half a year depending on Individual response	Hydroxychloroquine	FVC; DLCO	6 months	None
Lepri et al. 2016	CTD-ILD	Case series	21	Cumulative mean dose: SYN: 1.91 g; SSc: 1.75 g; MCTD: 1.4 g	2 years	ASS: Azathioprine* 8, MTX*1, IVIG*1, cyclosporine*1, CYC*3; SSc: MTX*9, MMF*1 MCTD: MMF, CYC, MTX	FVC; DLCO	2 years	1 Arrhythmia; 3 fatigue; 8 infections (2 serious with hospitalization)
Sharp et al. 2016	CTD-ILD	Case series	24	One cycle: IV, 1000 mg administered on day 0 and day 14	1–2 cycles	Oral immunosuppression	FVC; DLCO	6–12 months	None
Daoussis et al. 2017	SSc-ILD	Cohort (prospective)	33	One cycle: IV, 375 mg/ m^2^, once weekly*4 weeks, every 6 months	≥ 2 cycles	MTX*2; Hydroxychloro-quine*1; MMF*10	FVC; DLCO	2 years	2 Infusion reactions
Md Yusof et al. 2017	RA-ILD	Cohort (retrospective)	56	One cycle: IV, 1000 mg administered on day 1 and day 14	≥ 1 cycle	CYC	FVC; DLCO	6-12 months	12 Chest infections (none hospitalized)
Sari et al. 2017	SSc-ILD	Case series	14	One cycle: IV, 1000 or 500 mg, 2 infusions biweekly	4 received 1 cycle; 2 received 2 cycles; 2 received 3 cycles; 4 received 4 cycles; 2 received 5 cycles	None	FVC	15 months	None
Doyle et al. 2018	ASS-ILD	Case series	12	Not mentioned	Mean time to initiation of RTX after ILD identification: 4.4 years	Azathioprine, MMF, CYC, IVIG	FVC; DLCO	2 years	1 Anaphylaxis and 2 serious gastrointestinal complications requiring surgery (not described), but later resumed RTX
Sircar et al. 2018	SSc-ILD	RCT	30	One cycle: IV, 1000 mg administered on day 0 and day 15, every 6 months	2 cycles	None	FVC	6 months	1 Severe pulmonary arterial hypertension, 3 infusion reactions
Duarte et al. 2019	CTD-ILD	Case series	49	One cycle: IV, 1000 mg, 2 infusions biweekly	Median number of cycles was 2	None	FVC; DLCO	1 year	None
Ebata et al. 2019	SSc-ILD	Non-randomized study (retrospective)	9	One cycle: IV, 375 mg/ m.^2^, once weekly*4 weeks	1 up to 3 cycles	Maintenance therapy with immunosuppressant agents	FVC; DLCO	2 years	None
Fui et al. 2019	RA-ILD	Cohort (retrospective)	14	One cycle: IV, 1000 mg administered on day 0 and day 14	Treated for more than 1 year	None	FVC; DLCO	1 year	Discontinued in 2 patients with refractory severe arthritis

**Table 2:** Characteristics of studies included in the descriptive review (n = 6 studies)

Study name and year	CTD-ILD subtype	Type of study	N	RTX Dose	Duration	Concurrent immunotherapy	Outcomes	Clinical follow-up	Adverse events with RTX	Primary reason for qualitative review
Sem et al. 2009	ASS-ILD	Case series	11	One cycle: 2-IV, 375 mg/ m^2^, monthly; 8-IV, 1000 mg administered on day 0 and day 14; 1–IV, 700 mg administered on day 0 and day 14	1 cycle	None	FVC; DLCO	6 months	1 infusion reaction	Provided only percentage improvements
Marie et al. 2012	ASS-ILD	Case series	7	One cycle: IV, 1000 mg administered on day 0 and day 14	1 cycle and another IV 1 g after 6 months	None	FVC; DLCO	1 year	None	No standard deviation data before and after treatment
Allenbach et al. 2015	ASS-ILD	Case series	10	One cycle: IV, 1000 mg administered on day 0 and day 14	1 cycle and another IV 1 g after 6 months	IVIG	FVC; DLCO	1 year	6 infections (not hospitalized)	No specific data on PFT change
Andersson et al. 2015	ASS-ILD	Case series	24	2- IV, 375 mg/ m^2^, monthly; 21-IV, 1000 mg administered on day 0 and day 14; 1- reduced dose because of perceived infection risk	Median number of cycles was 2.7	AZA, CYC, MMF	FVC; DLCO	10–60 months	6 infections (not hospitalized) and 1 purpuric rash	No standard deviation data before and after treatment
Chartrand et al. 2016	CTD-ILD	Cohort (retrospective)	24	One cycle: 22-IV, 1,000 mg administered on day 0 and day 14; 2-IV, 375 mg/ m.^2^, once weekly*4 weeks	14 ≥ 1 cycles	MMF, CYC	FVC	≥ 2 years	1 infusion reaction	Provided FVC curves but no raw data values
Vadillo et al. 2020	RA-ILD	Cohort (prospective)	31	Not mentioned specifically	Median number of cycles was 3.4	None	FVC; DLCO	6 years	1 infection and 1 pancytopenia	Provided only mean difference values without calculatable SD

**Table 3:** Summary clinical findings for meta-analysis and descriptive review cohorts

Meta-analysis (N = 14 studies)				
Age (mean age ± SD)	Sex (F/M)	Disease type	ILD (fibrotic vs non-fibrotic)	Rationale for initiating RTX
52 ± 7	184/77 (not delineated in two studies (N = 35))	SSc = 120 RA = 98 ASS = 22 SS = 12 SLE = 7 IIM = 8 Unclassifiable = 4	Fibrotic = 290* OP = 3 Nodular or GGO = 4	Non-response to prior therapy = 260 Firstline = 3 Randomized = 33

Descriptive analysis (N = 6 studies)				
Age (mean age)	Sex (F/M)	Disease type	ILD (fibrotic vs non-fibrotic)	Rationale for initiating RTX
56 ± 3	41/34 (not delineated in one study (N = 31))	SSc = 3 RA = 39 ASS = 52 IIM = 3 MCTD = 2 Unclassifiable = 1	Fibrotic = 95* OP = 2 LIP = 1 Unclassified = 1	Non-response to prior therapy = 62 Firstline = 7 (Not delineated in 2 studies (N = 38))

*ASS*  antisynthetase syndrome, *F* female, *GGO*  ground glass opacities, *IIM* idiopathic inflammatory myopathy, *ILD* interstitial lung disease *LIP* lymphocytic interstitial pneumonia, *M* male, *MCTD* mixed connective tissue disease, *OP* organizing pneumonia, *RTX* rituximab, *RA* rheumatoid arthritis, *SLE* systemic lupus erythematosus, *SSc* scleroderma or systemic sclerosis, *SD* standard deviation

^*^Inclusive of mixed fibrotic and GGO infiltrates, usual interstitial pneumonia, nonspecific interstitial pneumonia (NSIP), and NSIP with organizing pneumonia

### Effect size of RTX on FVC% and DLCO% endpoints

**Fig. 2:**
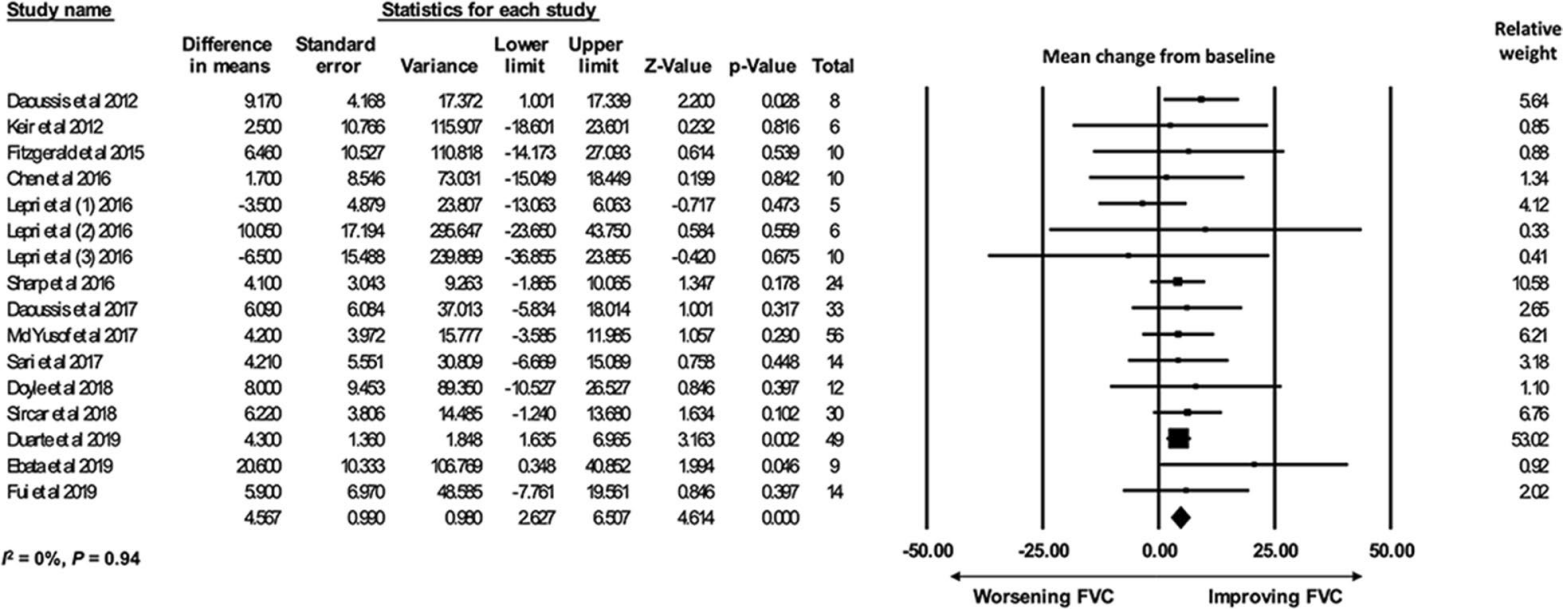
FVC% meta-analysis forest plot, random-effects analysis

**Fig. 3:**
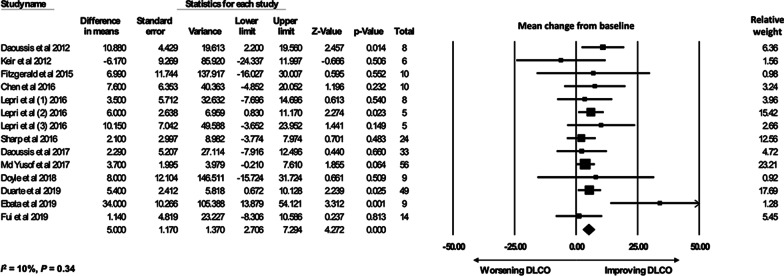
DLCO% meta-analysis forest plot, random effects analysis

A total of 296 CTD-ILD patients from 14 studies with available pre- and post-treatment FVC% contributed to the quantitative meta-analysis (*I*^*2*^ = 0%, *P* = 0.94). There was an increase in FVC% before and after RTX treatment with a MD of 4.57% [95% CI, 2.63–6.51] (Fig. 2). Quantitative meta-analysis for DLCO% included 246 patients from ten studies (*I*^*2*^ = 10%, *P* = 0.34). There was an increase in DLCO% (MD 5.0% [95% CI 2.71–7.29]) after RTX treatment (Fig. 3).

### Safety outcomes as reported for all studies

Infusion related reactions, including fever, chills, and rigors were the most reported adverse effects along with non-serious infections. Fifty six of 411 treated patients suffered some type of adverse effect (13.6%). As presented in Table 1, one study reported 12 non-serious chest infections[14] while eight studies reported zero events. There were no reported deaths as a direct result of RTX treatment.

### Risk of bias and publication bias assessment

**Fig. 4:**
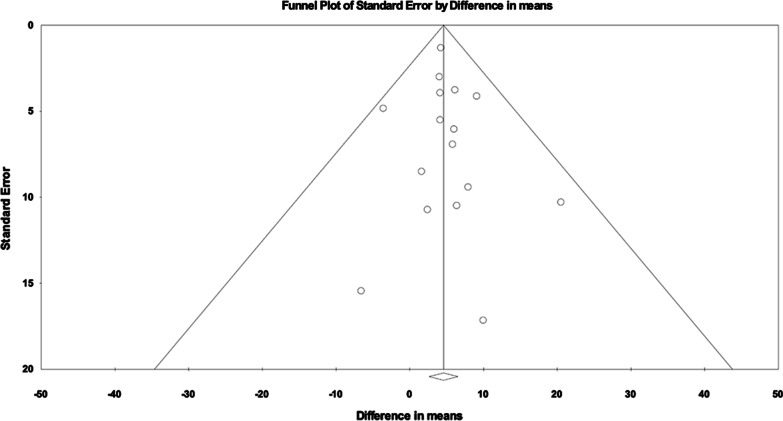
Funnel plot for FVC meta-analysis

**Fig. 5:**
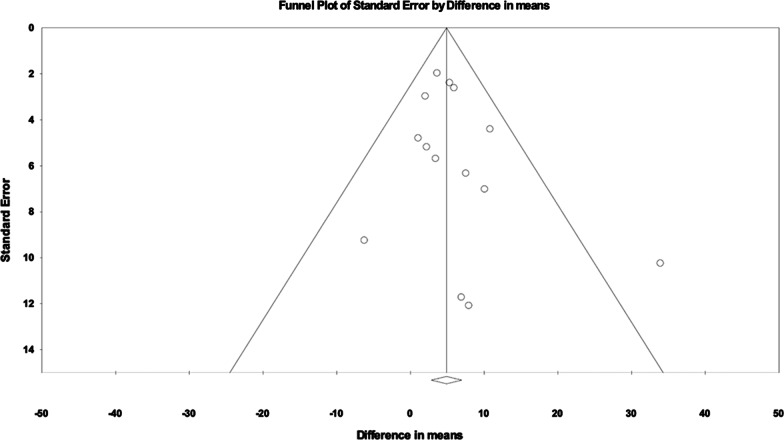
Funnel plot for DLCO meta-analysis

Systematic biases were scored for included studies and presented in Additional file 1: Table S2. A particular limitation for meta-analysis was the lack of RCT studies. As multiple reports with varied definitions of positive outcomes were available and our intent was to systematically review effect size, we included only those studies reporting specific baseline and post-treatment lung function. Many screened publications were also case reports with less than five patients, preemptively excluded due to smaller studies contributing greater bias. There did not appear to be publication bias for the two primary pulmonary function outcomes as represented by funnel plots (Figs. 4 and 5). The Egger’s regression asymmetry test demonstrated P values of 0.60 and 0.28 (P > 0.05 suggests no publication bias) for the outcomes of FVC% and DLCO%, respectively.

### Analysis of studies included in the descriptive review

Six studies with various PFT outcomes but unreported specific pre and post treatment FVC% and DLCO% (with related standard deviation required for pooled analysis) that could not be combined were analyzed descriptively (Table 2). A cohort of 14 CTD-ILD patients who received more than one cycle of RTX found FVC% trajectory increased in eight and declined in six [23]. Another RA-ILD study found RTX treatment appeared to lower the risk of respiratory impairment (defined as a decline ≥ 5% in the predicted FVC) compared to untreated historical controls (hazard ratio, 0.51 [95% CI, 0.31–0.85]) [18]. Four case series reported RTX therapy in ASS-ILD. Andersson et al. showed that median FVC% and DLCO% increased by 24% and 17% respectively in 24 patients on RTX treatment for a median number of 2.7 cycles [20]. A case series of seven patients found that after one year of RTX treatment, median FVC% increased from 66% [range: 35–76] to 74% [range: 57–108] (P = 0.04); and median DLCO% increased from 39% [range: 20–57] to 59% [range: 49–72] (P = 0.001) [27]. Neither study was included in the quantitative meta-analysis due to the absence of reported and non-calculatable standard deviations for each PFT outcome. Allenbach et al. summarized the FVC findings of ten patients after 1.5 cycles of RTX, showing FVC improvement in four, stability in five, and worsening in one [17]. Lastly, a final study involving ASS-ILD found that after one cycle RTX, six of eleven patients showed > 10% improvement in FVC% and 3 had > 15% improvement in DLCO% [31].

## Discussion

To our knowledge this is the first systematic review and meta-analysis to assess RTX effect size on FVC% (MD of 4.57%) and DLCO% (MD of 5.0%) in patients with CTD-ILD, reporting a low but not insignificant level of drug-related adverse effects (13.6% of the pooled cohort). Over 240 pooled patient observations were included for each functional endpoint suggesting RTX may modestly improve or stabilize lung function as an adjunct to traditional immunosuppression.

While the combined effect size on PFT outcomes for CTD-ILD was reported here, it is worth reviewing the individual characteristics and responses to RTX in reported specific diseases. SSc has the highest mortality associated with ILD [34] along with the highest ILD prevalence [35]. Incidentally, studies involving SSc-ILD patients were also the largest represented subtype in our meta-analysis. FVC% appeared to improve after RTX therapy in the combined meta-analysis [13, 15, 21, 22, 30] inclusive of the only RCT in our systematic review [19]. Divergent findings though were reported in 23 patients of a subgroup in the study by Lepri et al. [15]. Similarly, DLCO in SSc-ILD appeared to stabilize or improve with RTX treatment. The second largest group of represented CTD-ILD in our meta-analysis was ASS-ILD, whose disease-defining manifestations often include inflammatory myopathy, ILD, arthritis, and various hand manifestations [36, 37]. ILD prevalence ranges from 67 to 100% based on antibody type and the diagnostic criteria used [38, 39]. Our meta-analysis and descriptive review included five studies demonstrating FVC improvement or stability with RTX [17, 20, 26, 27, 31]. ILD is also an important comorbidity of RA often associated with similar outcomes to IPF, prompting novel approaches to treatment to improve or extend survival [40]. RTX has already been approved for the treatment of joint symptoms while there is less data on the treatment of related ILD. RTX demonstrated stabilization and in some cases, improvement of ILD in patients with RA [41]. All included studies in our meta-analysis suggested stabilization or improvement of FVC and DLCO [14, 18, 28]. ILD associated with primary Sjögren’s syndrome (pSS) occurs less commonly compared to other CTD though contributes to significant morbidity and mortality [42, 43]. RTX may be a promising treatment in this setting given the suggested role of B cell hyperactivity in the immunopathogenesis of pSS [44]. The study by Chen et al. in included in this meta-analysis suggests RTX may stabilize pulmonary function in patients with pSS [29].

Data for treatment of the other CTD-ILD with RTX remains limited. ILD prevalence in the idiopathic inflammatory myopathies (IIMs) is about 30–40% and contributes to an estimated mortality of 40% [45]. A recent systematic review suggested immunosuppressive therapies were associated with significant functional improvement for most patients with IIMs and chronic ILD, though the mortality of rapidly progressive disease remains high [46]. A case report of four patients on RTX therapy for rapidly progressive lung disease related to anti-MDA5 antibody-positive amyopathic dermatomyositis showed clinically significant improvement in lung function, though post-treatment infection risk was increased [47]. ILD is less common in systemic lupus erythematosus (SLE). A large multicenter observational cohort of 147 patients suggested RTX may be a possible maintenance option [48], though little data was provided regarding response of ILD findings to directed treatment. In contrast, there are reports of rituximab-induced interstitial pneumonitis seen in SLE patients [49].

Pooled analysis across a spectrum of CTD-ILD suggested a modest 4–5% increase in both FVC% and DLCO% after treatment with RTX compared to stabilization or slowing of prior decline. Similar effect in improved PFT findings were seen in prior observational and RCTs assessing CYC and MMF in patients with scleroderma-ILD [50–53], as well as azathioprine in one series of CTD patients with fibrotic ILD [54]. Specific effect sizes ranged from 1.5% to 15% in terms of FVC% change. In the majority of included studies for this meta-analysis, patients were considered non-responsive or refractory to typical immunosuppression, suggesting a separate role for the targeting of other immune-mediated or inflammatory processes for RTX. Current approval of anti-fibrotic therapy for progressive fibrotic lung disease including CTD-ILD warrants consideration as preferred secondary or tertiary therapies for lung fibrosis over RTX [55, 56]. Whether anti-fibrotics are preferred over RTX remains debatable but might be more justified by available controlled studies in progressive ILD over currently uncontrolled and mostly descriptive data for RTX [57]. Lack of controlled data though does not necessarily suggest evidence of inefficacy with future controlled studies needed to support or refute the role of RTX. Relevant decision making for clinicians and patients therefore may be geared more towards balancing quality of life vs. adverse effects. It is unclear from our meta-analyses if increases in FVC% or DLCO% of 4 to 5% compared to pre-treatment baseline is clinically impactful (in terms of symptomatic or radiologic improvement) or sustained with subsequent treatment. These remain important caveats to real-world management as RTX immunosuppression is often more prolonged and less immediately reversible. One study reported the direct effects of immunosuppressant treatment (not RTX) on patient-reported outcomes (PRO) and health-related quality of life in scleroderma-ILD patients, noting improvements in PRO scores meeting minimal clinically important differences, but little correlation with baseline or subsequent FVC change [58].

Additional concerns include cost and risk of serious adverse effects which may limit immediate or first-line use of RTX in the treatment of CTD-ILD. Our systematic review suggests RTX was overall well-tolerated and safe in the majority of treated CTD-ILD patients [59], including those on long-term therapy [60]. RTX-associated interstitial lung disease (RTX-ILD) or lung injury may be particularly concerning in those with already present lung disease. However, RTX-related lung injury was previously reported more commonly in combination with other chemotherapeutic agents for the treatment of lymphoma, which may confound accurate assessments of causation [61]. No direct RTX-related ILD or lung injury was reported in our review, highlighted by only a few serious adverse events due to infection with no therapy-related deaths.

There are several limitations to our systematic review and meta-analysis. First, variation in disease subtype and patient characteristics likely increased pooled heterogeneity and limits a true assessment of treatment effect size. We accounted for this with use of a random effects model and estimated the degree of heterogeneity for each endpoint, though still found *I*^2^ for example in the quantitative meta-analysis of FVC (*I*^2^ = 0%) was low and suggestive of little heterogeneity. It is known though that *I*^2^ does not necessarily describe how much an effect size varies but more what proportion of the observed variance would remain if all sampling error could be eliminated. When *I*^*2*^ is near zero dispersion in a forest plot may be minimal but does not suggest the absence of any heterogeneity, particularly when sample sizes in included studies were small with wider standard variations [62]. Additional limitations to our meta-analysis include the inability to account for duration of drug exposure, variation in timing of PFT follow-up, and the balance of CTD-ILD subtypes, of which pooled analyses may be weighed by one disease type over another. As presented in Tables 1 and 3, patients treated with RTX were often treated after or concomitantly with other immunosuppressive agents. CYC has previously demonstrated short-term improvement in FVC in SSc-ILD patients, though with a higher incidence of adverse effects [50, 63]. AZA as maintenance therapy after six months of CYC did not demonstrate significant FVC improvement in this same disease subtype [64]. MMF is thought to be safer and equally effective in the management of CTD-ILD when compared to CYC and AZA [50, 54]. We could not completely account for the role of concomitant therapy which may have also contributed to measured effect sizes. Lastly, pulmonary hypertension is common and well-described in CTD-ILD [65, 66]. Unfortunately, no included studies in our meta-analysis provided descriptions or assessments of pulmonary hypertension as assessed either by echocardiography or right heart catheterization. Its presence particularly in more severe disease may confound DLCO% measurements and degree of suggested response to treatment.

## Conclusion

The present systematic review and meta-analysis suggests a modest stabilization or improvement in pulmonary function (FVC% and DLCO%) in CTD-ILD patients treated with RTX. There also appears to be a relatively safe adverse effects profile alone or in combination with other immunosuppressant agents. However, the lack of RCTs and other controlled quantitative studies along with heterogeneity of underlying diseases when data is pooled may pose important limitations for the confident use of RTX in real-world practice, with treatment initiation considered on a case-by-case basis.

## Supplementary Information

**Additional file 1: Table S1.** Results of search terms and strategies. **Table S2.** Risk of biasassessment.

## Data Availability

All data generated or analyzed during this study are included in this published article and its supplementary information files.

## Abbreviations

ASS: Antisynthetase syndrome
AZA: Azathioprine
CI: Confidence interval
CTD: Connective-tissue disease
CTD-ILD: Connective-tissue disease-related interstitial lung disease
CYC: Cyclophosphamide
DLCO%: Percent predicted diffusion capacity for carbon monoxide
FVC%: Percent predicted forced vital capacity
IIM: Idiopathic interstitial pneumonia
ILD: Interstitial lung disease
JBI: Joanna Briggs Institute
MMF: Mycophenolate mofetil
PFT: Pulmonary function test
PRISMA: Preferred Reporting Items for Systematic reviews and Meta-Analyses
pSS: Primary Sjögren’s syndrome
RA: Rheumatoid arthritis
RTX: Rituximab
SSc: Systemic sclerosis
SLE: Systemic lupus erythematosus
WMD: Weighted mean difference
