# Supplementary material for: Effect size of rituximab on pulmonary function in the treatment of connective-tissue disease-related interstitial lung disease: a systematic review and meta-analysis
Source: Respir Res. 2022 Jun 21;23:164. doi: 10.1186/s12931-022-02082-x (PMC9215101; doi:10.1186/s12931-022-02082-x)
Supplement: Supplementary file 1 — Additional file 1: Table S1. Results of search terms and strategies. Table S2. Risk of biasassessment. [file 12931_2022_2082_MOESM1_ESM.docx]

**Table S1) Results of search terms and strategies**

**Pubmed: N = 3509 (non-duplicate)**

| Search Strategy | Results |
| --- | --- |
| (rituximab) AND (connective-tissue disease-related interstitial lung disease) | 4 |
| (rituximab) AND (interstitial lung disease) | 583 |
| (rituximab) AND (systemic sclerosis) | 225 |
| (rituximab) AND (Sjogren) | 100 |
| (rituximab) AND (mixed connective tissue disease) | 28 |
| (rituximab) AND (antisynthetase) | 46 |
| (rituximab) AND (anti synthetase) | 37 |
| (rituximab) AND (anti-Jo1) | 8 |
| (rituximab) AND (idiopathic inflammatory myopathy) | 269 |
| (rituximab) AND (myositis) | 263 |
| (rituximab) AND (rheumatoid arthritis related interstitial lung disease) | 17 |
| (rituximab) AND (rheumatoid arthritis) | 1907 |
| (rituximab) AND (systemic lupus erythematosus) AND (interstitial lung disease) | 22 |

**Cochrane Library: N=91 (non-duplicate)**

| Search Strategy | Results |
| --- | --- |
| (rituximab) AND (connective-tissue disease-related interstitial lung disease) | 1 |
| (rituximab) AND (interstitial lung disease) | 5 |
| (rituximab) AND (systemic sclerosis) | 2 |
| (rituximab) AND (Sjogren) | 10 |
| (rituximab) AND (mixed connective tissue disease) | 1 |
| (rituximab) AND (antisynthetase) | 1 |
| (rituximab) AND (idiopathic inflammatory myopathy) | 2 |
| (rituximab) AND (myositis) | 4 |
| (rituximab) AND (rheumatoid arthritis) | 40 |
| (rituximab) AND (systemic lupus erythematosus) | 25 |

**Embase: N=206 (non-duplicate)**

| Search Strategy | Results |
| --- | --- |
| #1: (rituximab [Title/Abstract]) AND (forced vital capacity [Title/Abstract]) | 80 |
| #2: (rituximab [Title/Abstract]) AND (fvc [Title/Abstract]) | 128 |
| #3: (rituximab [Title/Abstract]) AND (lung function [Title/Abstract]) | 77 |
| #3: (rituximab [Title/Abstract]) AND (pulmonary function [Title/Abstract]) | 115 |

**Table S2) Risk of bias assessment**

| Number | Study name | Study type | Assessment tool | First reviewer assessment | Second reviewer  assessment | Consensus discussion required? (Yes or No) |
| --- | --- | --- | --- | --- | --- | --- |
| 1 | Daoussis et al. 2017 | Cohort (prospective) | JBI Critical Appraisal Checklist for Cohort Studies | 11*Yes | 11*Yes | No |
| 2 | Sari et al. 2017 | Case Series | JBI Critical Appraisal Checklist for Case Series | 10*Yes | 10*Yes | No |
| 3 | Ebata et al. 2019 | non-randomized controlled study (retrospective) | JBI Critical Appraisal Checklist for Quasi-Experimental Studies (non-randomized experimental studies) | 8*Yes  1* Unclear | 9*Yes | No |
| 4 | Keir et al. 2012 | Case Series | JBI Critical Appraisal Checklist for Case Series | 10*Yes | 8*Yes  1*Unclear  1*No | Yes |
| 5 | Fitzgerald et al. 2015 | Case Series | JBI Critical Appraisal Checklist for Case Series | 9*Yes  1*No | 9*Yes  1*Unclear | No |
| 6 | Lepri et al. 2016 | Case Series | JBI Critical Appraisal Checklist for Case Series | 9*Yes  1*No | 7*Yes  2*Unclear  1*No | Yes |
| 7 | Doyle et al. 2018 | Case Series | JBI Critical Appraisal Checklist for Case Series | 10*Yes | 10*Yes | No |
| 8 | Md Yusof et al. 2017 | Cohort (retrospective) | JBI Critical Appraisal Checklist for Cohort Studies | 9*Yes  2*No | 10*Yes | No |
| 9 | Fui et al. 2019 | Cohort (retrospective) | JBI Critical Appraisal Checklist for Cohort Studies | 9*Yes  2*No | 7*Yes  3*No  1*Unclear | Yes |
| 10 | Chen et al. 2016 | Case Series | JBI Critical Appraisal Checklist for Case Series | 9*Yes  1*No | 8*Yes  2*Unclear | Yes |
| 11 | Daoussis et al. 2012 | Case Series | JBI Critical Appraisal Checklist for Case Series | 10*Yes | 7*Yes  2*Unclear  1*No | Yes |
| 12 | Sircar et al. 2018 | RCT | the Cochrane risk of bias tool for randomized trials | See below* | See below* | No |
| 13 | Chartrand et al. 2016 | Cohort (retrospective) | JBI Critical Appraisal Checklist for Cohort Studies | 7*Yes  3*NG  1*No | 10*Yes | Yes |
| 14 | Sharp et al. 2016 | Case Series | JBI Critical Appraisal Checklist for Case Series | 10*Yes | 8*Yes  2*Unclear | Yes |
| 15 | Duarte et al. 2019 | Case Series | JBI Critical Appraisal Checklist for Case Series | 8*Yes  2*Unclear | 7*Yes  3*Unclear | Yes |
| 16 | Andersson et al. 2015 | Case Series | JBI Critical Appraisal Checklist for Case Series | 10*Yes | 10*Yes | No |
| 17 | Marie et al. 2012 | Case Series | JBI Critical Appraisal Checklist for Case Series | 10*Yes | 8*Yes  2*Unclear | Yes |
| 18 | Vadillo et al. 2020 | Cohort (prospective) | JBI Critical Appraisal Checklist for Cohort Studies | 11*Yes | 11*Yes | No |
| 19 | Allenbach et al. 2015 | Case Series | JBI Critical Appraisal Checklist for Case Series | 10*Yes | 10*Yes | No |
| 20 | Sem et al. 2019 | Case Series | JBI Critical Appraisal Checklist for Case Series | 10*Yes | 8*Yes  2*Unclear | Yes |

**Results of Cochrane risk of bias tool for randomized trials used for Sircar et al. 2018**

| Risk of bias | First reviewer assessment | Second reviewer assessment |
| --- | --- | --- |
| Random sequence generation | Low risk | Low risk |
| Allocation concealment | Low risk | Low risk |
| Blinding of participants and personnel | Unclear risk | High risk |
| Blinding of outcome assessment | High risk | Unclear risk |
| Incomplete outcome data | Low risk | Low risk |
| Selective reporting | Low risk | Unclear risk |
| Others | Low risk | Low risk |
| Consensus discussion? (Yes or No) | Yes | |
